# Gangrenous Cholecystitis Secondary to Pneumatosis Intestinalis and Portal Venous Gas: A Case Report

**DOI:** 10.7759/cureus.71128

**Published:** 2024-10-09

**Authors:** Kei Harada, Takahisa Fujikawa, Yusuke Uemoto, Yuichiro Kawamura

**Affiliations:** 1 Surgery, Kokura Memorial Hospital, Kitakyushu, JPN

**Keywords:** bowel ischemia, gangrenous cholecystitis, pneumatosis intestinalis, portal venous gas, sepsis

## Abstract

Pneumatosis intestinalis (PI) and portal venous gas (PVG) are pathological conditions suggesting serious underlying diseases such as intestinal ischemia, intestinal wall infarction, and necrotizing enterocolitis. Therefore, early detection, comprehensive management, and timely treatment of the underlying disease are important for improving outcomes. We experienced a case of a patient who developed gangrenous cholecystitis secondary to PI and PVG. In this case, it was suggested that gangrenous cholecystitis may have been induced by retrograde infection due to the proliferation of intestinal bacteria and increased intestinal pressure caused by the reduced intestinal peristalsis due to PI and PVG. Herein, we report the successful treatment of this case with a literature review.

## Introduction

Pneumatosis intestinalis (PI) is a condition characterized by the presence of gas within the walls of the gastrointestinal (GI) tract and has a clinical course that varies depending on the underlying disease and its severity [1, 2]. Similarly, portal venous gas (PVG) is a rare condition associated with a poor prognosis, with PI and PVG sometimes coexisting and frequently cited as indicators of severe outcomes [3, 4].

When imaging technology was not widely available, the discovery of PI and PVG was assumed to be due to intestinal ischemia, and historically exploratory laparotomy was required. However, with the improvement in the sensitivity of computed tomography (CT) scans and the development of clinical algorithms, there is an increasing number of reports of cases treated without exploratory laparotomy [5-7]. When PI and PVG are identified, treatment options often depend on the patient's clinical findings, imaging and laboratory findings, and the judgment of the treating physician, and treatment strategies remain controversial [5, 8].

We experienced a case of gangrenous cholecystitis, a rare complication in a patient with PI and PVG, and with strict management, the patient had a favorable clinical course. Herein, we report this case with a literature review, which may contribute to proposing future treatment strategies for PI and PVG.

## Case presentation

A 70-year-old man visited our hospital with the main complaint of sudden vomiting. He had a two-year medical history of type 2 diabetes mellitus with medication, hypertension, and a right above-knee amputation due to right lower extremity arteriosclerosis. He was taking antihypertensive drugs, oral hypoglycemic agents, and intestinal regulators. The oral hypoglycemic agent was a sodium-glucose cotransporter 2 (SGLT2) inhibitor. In addition, he was allergic to iopamidol. The vital signs recorded were as follows: blood pressure, 103/63 mm Hg; pulse rate, 84 beats/min; and body temperature, 36.2°C. The abdomen was soft and not distended, but was tender to palpation, most notably at the umbilicus. Bowel sounds were decreased. There was no guarding or rebound tenderness. The initial laboratory findings are shown in Table 1.

**Table 1:** Initial laboratory findings CRP, C-reactive protein; WBC, white blood cell counts; BUN, blood urea nitrogen; AST, aspartate transaminase; ALT, alanine transaminase; T-Bil, total-bilirubin; γ-GTP, γ-glutamyl transpeptidase; ALP, alkaline phosphatase; PT, prothrombin time activity; APTT, activated partial thromboplastin time; FDP, fibrinogen degradation products; HbA1c, hemoglobin A1c

Parameter	Result	Reference Range
CRP	34.5 mg/L	0.0-0.14 mg/L
WBC	21.3 × 10^3^/uL	3.3-8.6 × 10^3^/uL
BUN	53.5 mg/dL	8.0-20.0 × 10^3^/uL
Creatinine	3.01 mg/dL	0.65-1.0 mg/dL
Hemoglobin	12.9 g/dL	13.7-16.8 g/dL
Platelet	48.8 × 10^3^/μL	15.8-34.8 × 10^3^/μL
AST	236 U/L	13-30 U/L
ALT	173 U/L	10-30 U/L
T-Bil	4.3 mg/dL	0.4-1.5 mg/dL
γ-GTP	232 IU/L	<50 IU/L
ALP	518 U/L	38-113 U/L
PT	45%	70%-130%
APTT	48.6 seconds	24-39 seconds
D-dimer	10.3 ng/mL	<1.0 ng/mL
FDP	18.7 μg/mL	<5.0 μg/mL

**Figure 1:**
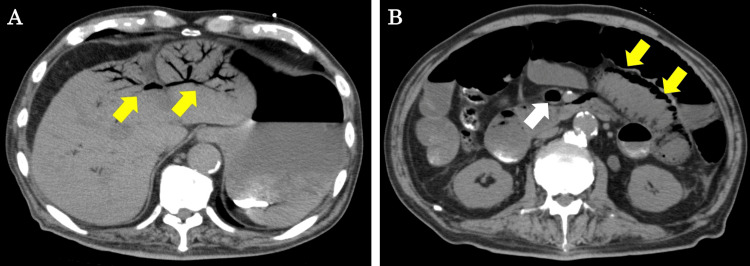
CT findings at initial examination (A) PVG can be observed mainly in the left lobe of the liver (yellow arrows). (B) Superior mesenteric venous gas (white arrow) and PI (yellow arrows) can be observed mainly in the proximal jejunum. CT, computed tomography; PVG, portal venous gas; PI, pneumatosis intestinalis

CT scan without contrast showed PVG reaching the liver surface, mainly in the left lobe of the liver (Figure 1A). Furthermore, superior mesenteric venous gas and PI were observed, mainly in the proximal jejunum (Figure 1B).

Based on these findings, we determined that there was a high possibility that the patient had multiple organ failure (MOF) due to intestinal necrosis or nonocclusive intestinal ischemia (NOMI). We immediately performed an emergency exploratory laparotomy. As a result, no findings suggestive of intestinal necrosis or NOMI were found. Observation of the intestines revealed normal intestinal peristalsis and no localized temperature drop or black or purple changes in the intestinal wall. Furthermore, intestinal blood flow was evaluated using indocyanine green (ICG) fluorescence, but no problems were found. After surgery, the patient was extubated and admitted to the intensive care unit (ICU). Conservative treatment, including food restriction during the acute postoperative period, total parenteral nutrition (TPN), and antibiotic therapy with carbapenem, was started. The progress of conservative treatment was generally good, but on postoperative day (POD) 6, the patient developed fever and increased inflammatory reaction. The patient had no symptoms other than fever, and abdominal examination findings were within normal limits. Furthermore, no bacteria were detected in blood cultures taken at the time of initial admission. The laboratory findings are shown in Table 2.

**Table 2:** Laboratory findings on POD 6 POD, postoperative day; CRP, C-reactive protein; WBC, white blood cell counts; BUN, blood urea nitrogen; AST, aspartate transaminase; ALT, alanine transaminase; T-Bil, total-bilirubin; γ-GTP, γ-glutamyl transpeptidase; ALP, alkaline phosphatase; PT, prothrombin time activity; APTT, activated partial thromboplastin time; FDP, fibrinogen degradation products

Parameter	Result	Reference Range
CRP	5.1 mg/L	0.0-0.14 mg/L
WBC	28.0 × 10^3^/uL	3.3-8.6 × 10^3^/uL
BUN	30.1 mg/dL	8.0-20.0 × 10^3^/uL
Creatinine	1.1 mg/dL	0.65-1.0 mg/dL
Hemoglobin	8.8 g/dL	13.7-16.8 g/dL
Platelet	43.2 × 10^3^/μL	15.8-34.8 × 10^3^/μL
AST	139 U/L	13-30 U/L
ALT	197 U/L	10-30 U/L
T-Bil	2.7 mg/dL	0.4-1.5 mg/dL
γ-GTP	263 IU/L	<50 IU/L
ALP	826 U/L	38-113 U/L
PT	53%	70%-130%
APTT	40.3 seconds	24-39 seconds
D-dimer	9.6 ng/mL	<1.0 ng/mL
FDP	16.5 μg/mL	<5.0 μg/mL

**Figure 2:**
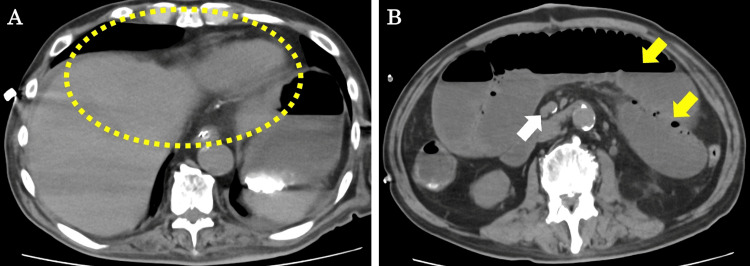
CT findings on POD 6 (A) PVG has disappeared (yellow dotted lines). (B) Superior mesenteric venous gas (white arrow) and PI have disappeared (yellow arrows). CT, computed tomography; POD, postoperative day; PVG, portal venous gas; PI, pneumatosis intestinalis

CT scan showed that the PVG had disappeared (Figure 2A), and although there was paralytic ileus, there were no findings suggestive of PI (Figure 2B).

**Figure 3:**
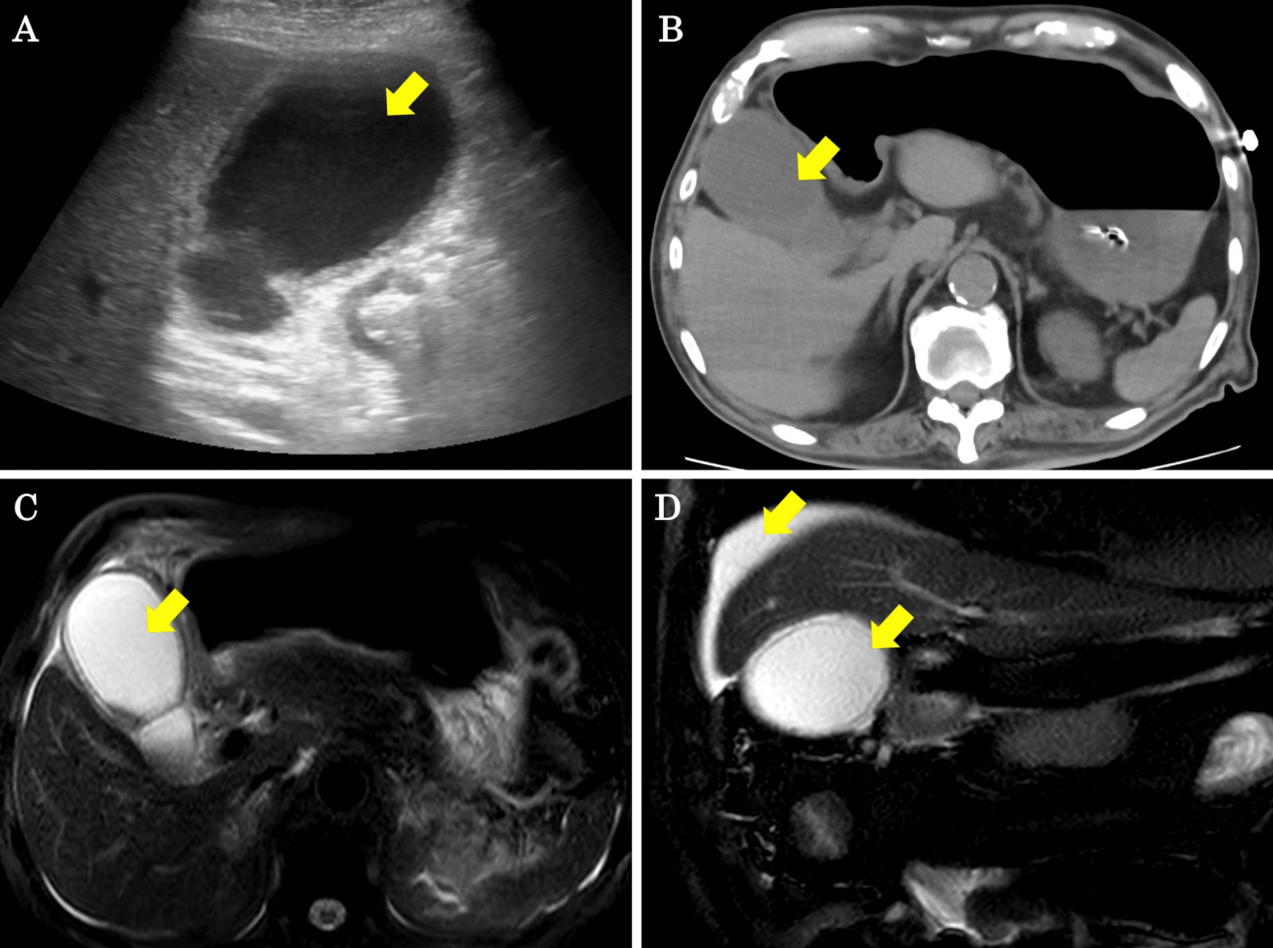
Abdominal ultrasound, CT, and MRCP findings on POD 6 (A, B) Abdominal ultrasound and CT show gallbladder enlargement, thickening of the gallbladder wall, and gallbladder debris (yellow arrows). (C, D) MRCP shows fluid accumulation around the gallbladder and on the surface of the liver, as well as edema of the gallbladder wall (yellow arrows). CT, computed tomography; POD, postoperative day; MRCP, magnetic resonance cholangiopancreatography

Abdominal ultrasound showed gallbladder enlargement, thickening of the gallbladder wall, and gallbladder debris (Figure 3A). CT scan showed inflammation around the gallbladder, gallbladder enlargement, and gallbladder wall thickening (Figure 3B). Magnetic resonance cholangiopancreatography (MRCP) showed fluid accumulation around the gallbladder and on the surface of the liver, as well as edema of the gallbladder wall (Figure 3C, 3D).

**Figure 4:**
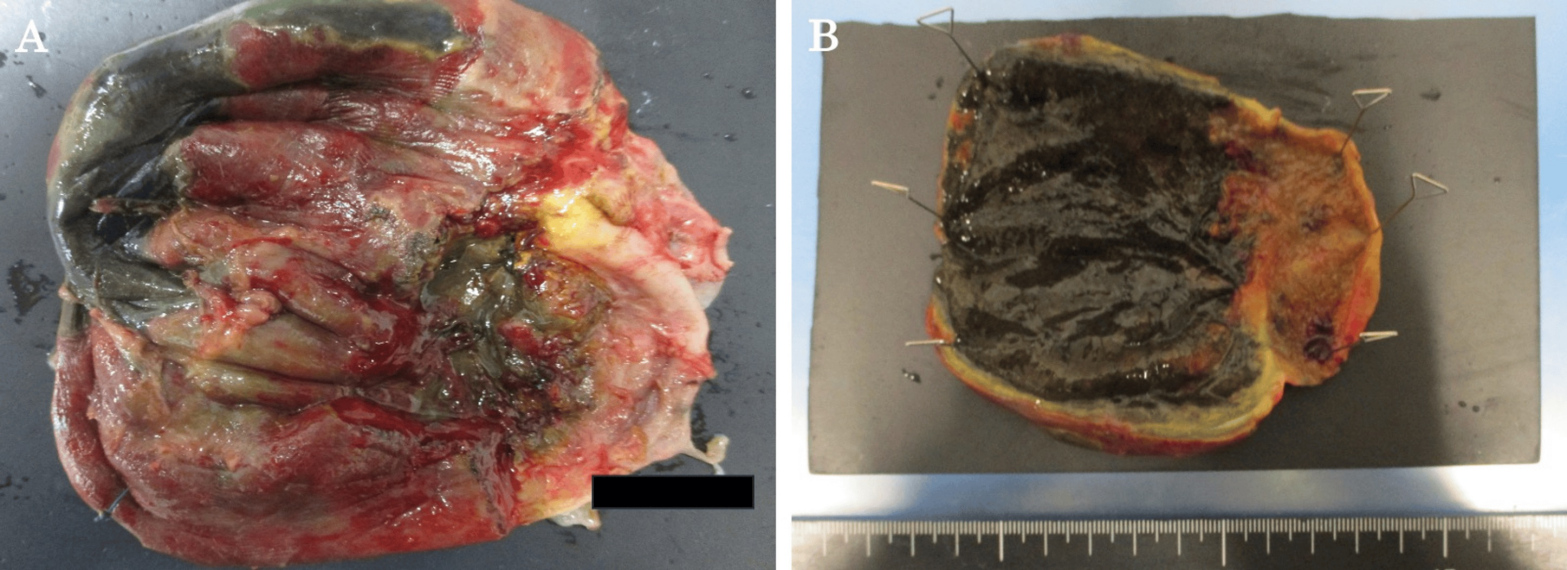
A specimen of the resected gallbladder (A, B) Findings of gangrenous cholecystitis. Thickening of the gallbladder wall and gallbladder necrosis are observed.

We diagnosed the patient with acute cholecystitis and performed an emergency open cholecystectomy. At surgery, the gallbladder was grossly enlarged and the gallbladder wall was black with necrosis. The bile in the gallbladder revealed a purulent appearance. The infected bile was submitted for bacterial culture, which revealed *Enterococcus faecium*. The resected gallbladder wall showed full-thickness necrosis, chronic active inflammatory cell infiltration, hemorrhage, small vessel infarction, and hemosiderin deposition, but no malignant findings were found. In addition, there were no stones in the resected gallbladder. These findings were consistent with those of gangrenous cholecystitis (Figure 4).

Although the patient subsequently developed perioperative pneumonia and drug-induced liver injury, the postoperative course was generally good, and the patient was discharged on POD 55 from the initial surgery. In reporting the case, we respected ethical considerations, including protection of personal privacy, and provided sufficient informed consent to the patient.

## Discussion

PI is a condition characterized by the formation of vacuoles of varying diameters, primarily in the submucosal and subserosa layers of the intestinal wall from a pathological perspective [2]. PVG is a rare radiological abnormality that is defined as a branching radiolucency on an X-ray or CT scan within 2 cm under the liver capsule [9]. PI and PVG are more likely to happen to people who have intestinal ischemia, severe GI infections, inflammatory diseases such as inflammatory bowel disease (IBD), traumatic or medically caused injuries, substance abuse, and long-term diseases such as chronic obstructive pulmonary disease (COPD) [10]. The presence of PVG with PI has been reported to be associated with a high mortality rate, reported to be 49% with PI and 29% without PI [7, 11]. In addition, the mortality rate for patients with PI and PVG resulting from bowel ischemia is high, frequently exceeding 50%, especially when accompanied by severe metabolic derangements and MOF [12]. Cases in which PI and PVG develop transiently due to mucosal damage such as transient ischemia are generally said to have a good prognosis [7]. If intestinal necrosis is present behind the development of PI and PVG, the clinical outcome is significantly poorer [3]. Diagnosis of intestinal necrosis is difficult, and strict management is important whether conservative treatment or exploratory laparotomy is performed.

This case is considered valuable in that it followed a good clinical course due to early detection and treatment of gangrenous cholecystitis, a rare complication of PI and PVG not caused by intestinal necrosis. Gangrenous cholecystitis is considered a more severe form of acute cholecystitis, which is primarily caused by vascular injury secondary to epithelial damage caused by persistent obstruction of the cystic duct [13]. Elderly people and people with diabetes are at higher risk of gangrenous cholecystitis, but it has been reported that because elderly people do not often show typical clinical symptoms of acute cholecystitis, diagnosis is delayed and the condition is more likely to become severe [14, 15].

The cause of this case of PI and PVG is presumed to be the slowing of intestinal peristalsis due to diabetes-related autonomic neuropathy and the stagnation and retention of intestinal gas. The onset of cholecystitis may have been the coincidence of different pathological conditions, but it is possible that cholecystitis was induced by retrograde infection at the same time as the proliferation of intestinal bacteria and increased intestinal pressure due to PI. This is supported by the detection of *Enterococcus faecium* in the bile in the gallbladder submitted for bacterial culture during surgery. It is difficult to consider the relationship between gangrenous cholecystitis and PI and PVG based on only one case, but our results suggest that treatment of PI and PVG should be carried out carefully, taking into account the patient's overall condition, abdominal findings, and various test results.

## Conclusions

We experienced a case in which a patient developed gangrenous cholecystitis, a rare complication secondary to PI and PVG. Complication management can be challenging due to the fact that PI and PVG have variable clinical courses, and the choice of treatment frequently depends on the patient's findings and the attending physician's judgment. This successful case suggests that strict management and early detection and treatment of complications are important. It is considered necessary to continue conducting case reports and research to establish treatment strategies for PI and PVG, which may lead to serious outcomes.
